# Sirtuin 1 regulates mitochondrial function and immune homeostasis in respiratory syncytial virus infected dendritic cells

**DOI:** 10.1371/journal.ppat.1008319

**Published:** 2020-02-27

**Authors:** Srikanth Elesela, Susan B. Morris, Samanthi Narayanan, Surinder Kumar, David B. Lombard, Nicholas W. Lukacs

**Affiliations:** 1 Department of Pathology, Michigan Medicine, University of Michigan, Ann Arbor, Michigan, United States of America; 2 Mary H. Weiser Food Allergy Center, Michigan Medicine, University of Michigan, Ann Arbor, Michigan, United States of America; 3 Institute of Gerontology, Michigan Medicine, University of Michigan, Ann Arbor, Michigan, United States of America; St. Jude Children’s Research Hospital, UNITED STATES

## Abstract

Respiratory syncytial virus (RSV) is the major cause of lower respiratory tract infection in children worldwide. Sirtuin 1 (SIRT1), a NAD+ dependent deacetylase, has been associated with induction of autophagy, reprogramming cellular metabolism, and regulating immune mediators. In this study, we investigated the role of SIRT1 in bone marrow dendritic cell (BMDC) function during RSV infection. SIRT1 deficient (SIRT1 -/-) BMDC showed a defect in mitochondrial membrane potential (Δ⍦m) that worsens during RSV infection. This defect in Δ⍦m caused the generation of elevated levels of reactive oxygen species (ROS). Furthermore, the oxygen consumption rate (OCR) was reduced as assessed in Seahorse assays, coupled with lower levels of ATP in SIRT1-/- DC. These altered responses corresponded to altered innate cytokine responses in the SIRT1-/- DC in response to RSV infection. Reverse Phase Protein Array (RPPA) functional proteomics analyses of SIRT1-/- and WT BMDC during RSV infection identified a range of differentially regulated proteins involved in pathways that play a critical role in mitochondrial metabolism, autophagy, oxidative and ER stress, and DNA damage. We identified an essential enzyme, acetyl CoA carboxylase (ACC1), which plays a central role in fatty acid synthesis and had significantly increased expression in SIRT1-/- DC. Blockade of ACC1 resulted in metabolic reprogramming of BMDC that ameliorated mitochondrial dysfunction and reduced pathologic innate immune cytokines in DC. The altered DC responses attenuated Th2 and Th17 immunity allowing the appropriate generation of anti-viral Th1 responses both *in vitro* and *in vivo* during RSV infection thus reducing the enhanced pathogenic responses. Together, these studies identify pathways critical for appropriate DC function and innate immunity that depend on SIRT1-mediated regulation of metabolic processes.

## Author summary

These studies further demonstrate that regulation of dendritic cell metabolism is mediated by SIRT1, a critical cellular sensor that controls both cellular and mitochondrial protein activation that facilitates proper innate immune activation. The absence of SIRT1 in DC leads to inappropriate metabolic processes, including fatty acid synthesis through Acetyl Co A pathways, which enhances the pathogenic immune responses leading to inappropriately modified acquired immunity. The present studies have identified several modified pathways in the absence of SIRT1 that may be relevant targets for intervention to avoid altered innate immunity. Inhibiting acetyl Co A activation by blocking ACC1 with a specific inhibitor corrected the altered metabolic state and resulted in the more appropriate innate and acquired immune responses during RSV infection. By identifying specific metabolic pathways in this manner, new therapeutics for RSV may be identified and allow interruption of the pathogen immune responses.

## Introduction

Human Respiratory syncytial virus (RSV) is a single-stranded, negative-sense RNA virus that belongs to the Paramyxoviridae family. It is the leading cause of acute respiratory infection during early childhood and is associated with a significant morbidity and mortality among infants, the elderly, and patients with chronic respiratory diseases worldwide [1–3]. Infants hospitalized with a severe RSV infection are at a greater risk for developing allergic asthma and recurrent wheezing later in life [4, 5], which suggests that a chronic alteration of the pulmonary immune environment may occur after RSV infection. Elevated levels of the pro-inflammatory cytokines, IL-1, IL-6, and IL-17A, have been observed in respiratory aspirate samples from patients hospitalized with RSV infections [6–8]. Importantly, IL-17a production drives mucus hypersecretion, neutrophil infiltration, and the suppression of CD8+ T-cell responses in RSV infection [9, 10]. Ribavirin and Pavilizumab are approved drugs for the treatment of RSV infection, but only Pavilizumab is effectively used under specific conditions such as prematurity and is only efficacious as a prophylactic [11, 12]. No vaccine is currently approved for RSV infections [13]. Hence, the development of drugs to treat RSV infection is an unmet need.

During RSV infection, pulmonary DCs drive innate immune responses and dictate the T cell adaptive immune responses. Once activated, DCs migrate to the lung draining lymph nodes and orchestrate T cell specific responses through antigen presentation and pro-inflammatory cytokine secretion. Previous work in our laboratory [14, 15] and others [16] suggest that autophagy facilitates intracellular pathogen recognition, DC maturation, and proinflammatory cytokine production. RSV infects the host cell through membrane fusion that allows direct entry of its genetic material into the cytoplasm [14]; therefore DC activation depends on autophagy to mediate critical endosomic TLR responses and appropriate innate immune responses [17, 18]. Since innate immune responses and mitochondria function are intertwined, the metabolic reprogramming of DC could be a viable target for therapeutic interventions [19].

Sirtuins are NAD^+^-dependent class III histone deacetylases whose functions are intrinsically linked to cellular metabolism with a significant impact through AMPK mediated pathways. Recent studies indicate that Sirtuins are not only important energy status sensors but also protect cells against metabolic stresses [20]. There are seven mammalian sirtuin orthologues, sirtuin1-7, which are localized in distinct cellular compartments. Sirtuin 1 (SIRT1), SIRT2, SIRT6, and SIRT7 localize, in part, in the nucleus, where they deacetylate histones thereby epigenetically regulating gene expression [20]. SIRT1 is present in both the nucleus and the cytoplasm, and deacetylates a range of proteins that are vital for mitochondrial function, metabolism, DNA repair, oxidative stress, apoptosis, cell cycle, circadian rhythms, autophagy, and other core biological processes. [21]. Importantly, SIRT1 deacetylates and activates 3 key autophagy proteins, ATG5, ATG7 and ATG8/LC3 to promote autophagy [22]. Mitochondria orchestrate signaling and effector functions to enhance immune cell activation and antimicrobial defense, and trigger inflammation in response to cell and tissue damage in host immune responses [23]. Mitochondria are the powerhouses of the cell where the TCA cycle, fatty acid oxidation (FAO) and oxidative phosphorylation (OXPHOS) occur, as well as numerous biosynthetic and degradative reactions. PPARγ coactivator 1-α (PGC1α) is the common transcriptional coactivator of PPARα and PPARγ and regulates FAO, mitochondrial biogenesis, and respiration [24]. PGC1α activation is controlled through reversible lysine side chain hyperacetylation that is attenuated by the enzymatic activity of SIRT1 [25]. Thus, activation or deactivation of metabolic enzymes as a consequence of SIRT1-mediated deacetylation could be relevant for controlling relevant pathways for RSV regulation.

In this study, we demonstrate that RSV infection alters mitochondrial function, and absence of SIRT1 further alters mitochondrial function and metabolic phenotype, resulting in the induction of fatty acid synthesis and leading to dysregulation of innate and adaptive immunity. SIRT1 regulation of ACC1 (Acetyl CoA carboxylase 1) activation appears to be central in controlling DC function and therefore subsequent immune mediated pathology. Collectively, these findings expand our understanding of the mitochondrial mediated innate immune response during RSV infection and may contribute to therapeutic strategies to prevent chronic pathology.

## Results

### SIRT1 regulates mitochondrial membrane potential and redox homeostasis

**Fig 1:**
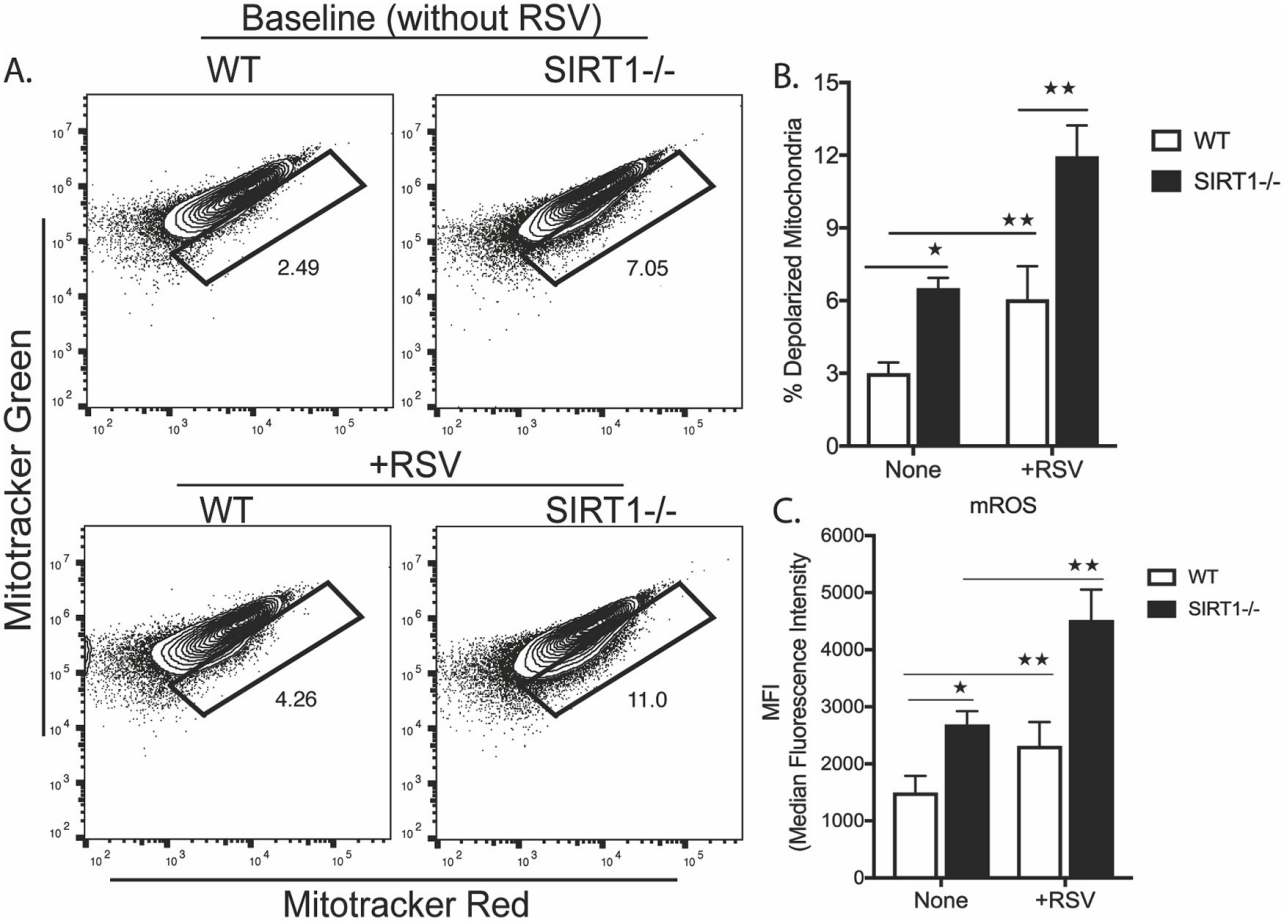
SIRT1 regulates mitochondrial membrane potential (Δ⍦*m*) and redox homeostasis. (A) Flow cytometry of WT or SIRT1-deficient BMDC with or without RSV infection using Mitotracker dyes. Mito Green (50nM) for mitochondrial mass and Mito Red (100nM) for membrane potential were used. (B) Bar graph showing percent depolarized mitochondria. (C) Bar graph showing mitochondrial ROS (mROS) determined using Mitosox (5uM) and analyzed with flow cytometer. Data are representative of at least 3 independent experiments. Values represent Mean ± SEM. **p< 0.005; *p< 0.05.

SIRT1 plays a critical role in cellular metabolism and specifically affects mitochondrial biogenesis and bioenergetics [25–27]. To examine the metabolic regulation during RSV infection, BMDC (Bone marrow derived dendritic cell) from C57BL/6J WT littermates (WT) and *Sirt1*^*f/f*^ CD11c-Cre (SIRT1-deficient, SIRT1-/-) mice were cultured with or without RSV infection and assessed for mitochondrial membrane potential (ΔΨm) using differential staining with mitotracker green and mitotracker red in live unfixed cells. Three group comparisons were used: WT vs WT+RSV; WT vs SIRT1 (Baseline, without RSV infection); and WT+RSV vs SIRT1+RSV. RSV infection in WT BMDC (WT+RSV) showed accumulation of dysfunctional/defective mitochondria as compared to uninfected WT BMDC. Thus, it appears that RSV interferes, directly or indirectly, with mitochondrial function. Uninfected SIRT1-/- BMDC showed a strikingly increased number of dysfunctional mitochondria as compared to the WT, even higher than WT+RSV. Furthermore, RSV infection in SIRT1-/- BMDC (SIRT1+RSV) resulted in accumulation of an extremely higher number of dysfunctional mitochondria as compared to WT+RSV (Fig 1A and 1B). The increased number of dysfunctional mitochondria could be due to an altered mitochondrial membrane potential that resulted from RSV infection and compounded by the absence of SIRT1.

Mitochondrial reactive oxygen species (mROS) are generated primarily in the electron transport chain and increase under conditions of impaired cellular or mitochondrial homeostasis [28]. As the defect in mitochondrial membrane can result in generation of mROS, we assessed mROS in WT and SIRT1-/- BMDC with and without RSV infection using flow cytometry based Mitosox staining. Elevated levels of mROS correlated with the increased number of dysfunctional mitochondria in SIRT1+RSV followed by SIRT1-/- and then WT+RSV (Fig 1C). Thus, the increased mROS corresponds to dysfunctional mitochondria.

### SIRT1 regulates mitochondrial respiration

**Fig 2:**
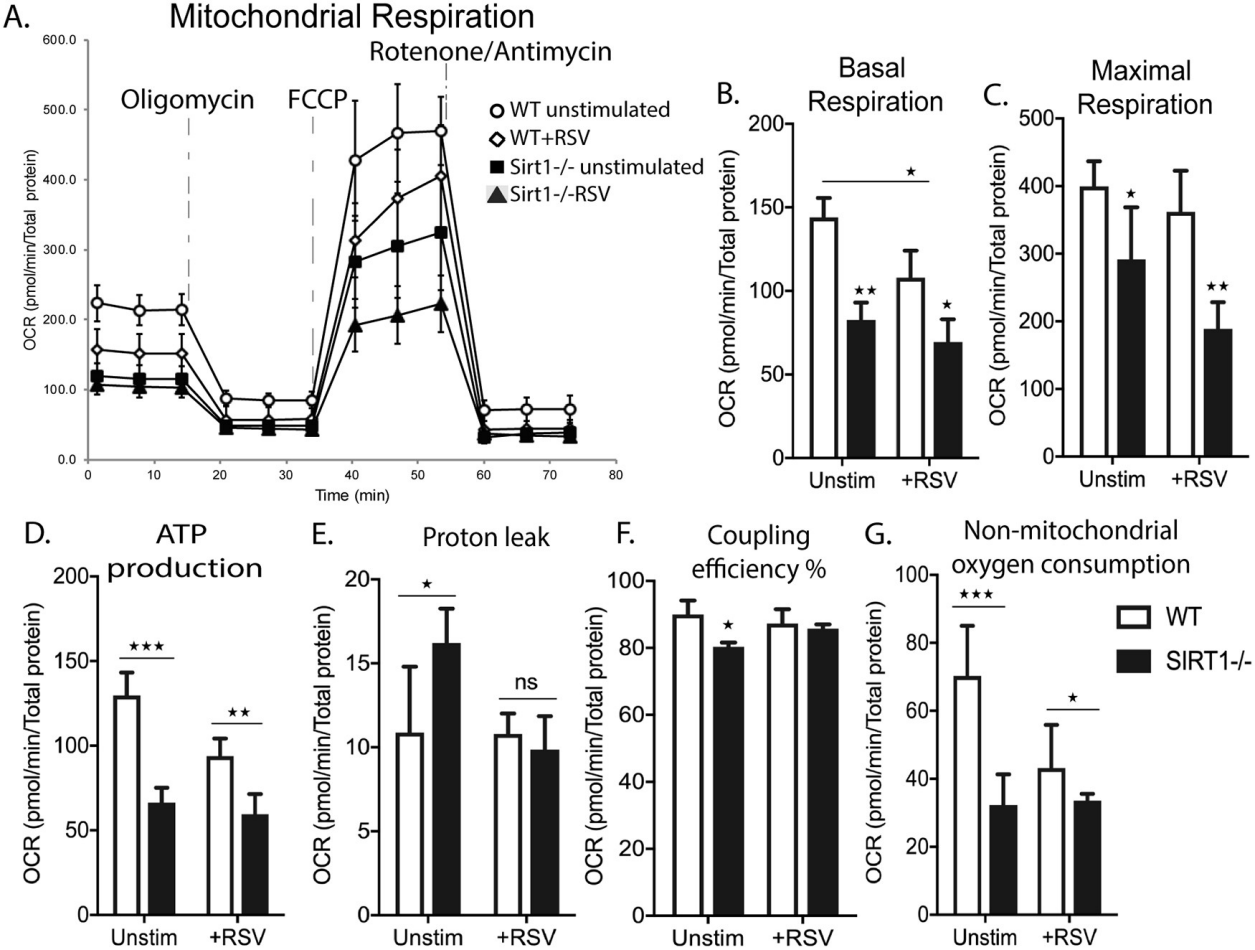
SIRT1 regulates mitochondrial respiration. BMDC from WT C57BL/6 and SIRT1-deficient mice were cultured for 10days in presence of GMCSF and infected with RSV MOI 2 for 2 hours. Mitochondrial respiration was determined using Cell Mito stress test kit and analyzed with Seahorse XFe96 analyzer. (A-C) Representative OCR of WT and SIRT1-deficient BMDC with or without RSV infection. (D-G) Representative ATP production, Proton leak, Spare respiratory capacity; Coupling efficiency (%) and Non-mitochondrial oxygen consumption was generated using Seahorse XF Cell Mito stress report generator. Data are representative of at least 3 independent experiments with at least three replicates per group. Values represent Mean ± SD. ***p< 0.0005 **p< 0.005 *p< 0.05.

Under normal conditions, energy homeostasis is largely dependent on mitochondria [18], as oxidative phosphorylation (OXPHOS), the chief source of ATP during cellular homeostasis in most cell types, occurs in mitochondria. WT and SIRT1-/- BMDC with and without RSV infection were assessed for oxygen consumption rate (OCR) using Seahorse based cell Mito Stress Test. RSV infection in WT BMDC demonstrated a significantly decreased basal OCR as compared to uninfected WT. At maximal respiration, when uncoupling of OXPHOS was initiated with FCCP, the OCR of WT+RSV remained significantly lower than the WT control. As expected, SIRT1-/- BMDC demonstrated a significantly decreased OCR at both, basal and maximal respiration, as compared to WT control and also as compared to WT+RSV. Interestingly, RSV infection in SIRT1-/- BMDC (SIRT1+RSV) did not lower OCR further as compared to uninfected SIRT1-/- BMDC (Fig 2A). Thus, it appears that absence of SIRT1 induces baseline dysfunction in mitochondria that continues during RSV infection. Decreased OCR is reflected in the impaired ATP generation in the respective groups (Fig 2D). Proton leak, Coupling Efficiency, and Non-mitochondrial O_2_ consumption were also determined (Fig 2E–2G) and demonstrated data consistent with the OCR. Mitochondrial dysfunction has been linked to decreased OCR in several studies [29]. In our present study, an increased number of dysfunctional mitochondria correlates with decreased OCR. However, no significant differences were observed in the glycolytic function of any these groups (S1 Fig). Together, these data demonstrate that SIRT1 regulates mitochondrial stability and function, and its absence could lead to metabolic reprogramming in DC that may alter the cellular immune response to RSV.

### SIRT1 mediated mitochondrial function regulates Innate and pathologic Th2 and Th17 immune response

**Fig 3:**
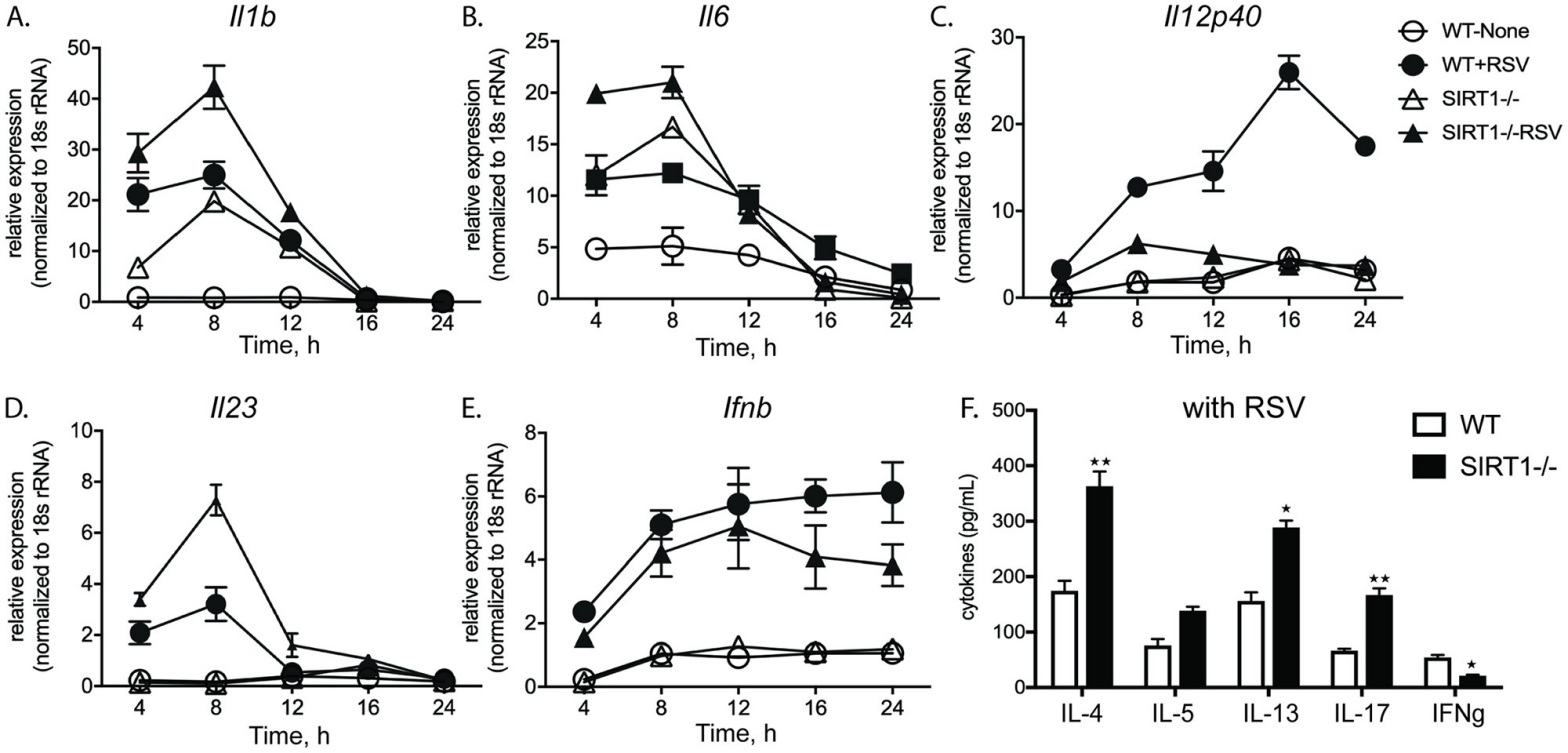
SIRT1 regulates innate and adaptive immune response. LDLNs from 8dpi RSV-infected WT mice were harvested and RSV responsive CD4+ T cells were isolated using Macs miltenyi CD4+ T cell isolation kit. Purified CD4+ T cells were then restimulated with BMDC pretreated with RSV (MOI 2) for 48h. (A-E) mRNA expression of WT and SIRT1-deficient BMDC infected with RSV for 8h (F) Cytokines secreted in 48h culture supernatants. Values are mean ± SEM. **p< 0.005 *p< 0.05.

Mitochondria, in addition to helping to fulfill cellular energy requirements, actively regulate innate immune responses against infections [30]. During metabolic stress, proteins from dysfunctional mitochondria released into the cytosol are directly recognized by the receptors of the innate immune system that orchestrates specific T cell responses. As it was evident that absence of SIRT1 induced baseline mitochondrial dysfunction and is further amplified during RSV infection we investigated the impact of dysfunctional mitochondria on innate and adaptive immune responses. WT and SIRT1-/- BMDC were treated with or without RSV infection, and innate cytokine gene expression was assessed over a 24 hr time course. RSV infection in WT BMDC significantly induced pro-inflammatory cytokines *Il-1β*, *Il-6* and *Il-23* as compared to uninfected WT. RSV infection of SIRT1-/- BMDC induced significantly higher *Il1β*, *Il6* and *Il23* expression early in the infection as compared to WT+RSV. However, RSV infection in SIRT1-deficient BMDC showed lower levels of the critical anti-viral *Il12* throughout the 24 hr time course (Fig 3A–3D). The expression of *Ifnβ* was similar in the earlier time points but by 24 hr post infection Sirt1-/- DC showed significantly less expression (Fig 3E). Increased *Il1β*, *Il6* and *Il23* at early time points can possibly prime T cell response towards increased Il17 leading to RSV induced inflammation and pathogenesis in SIRT1-deficient mice [15, 31].

To analyze T cell specific response, RSV responsive CD4+ T cells isolated from lung draining lymph nodes (LDLN) of 8day post RSV infected animals were restimulated with RSV treated BMDC, both SIRT1-/- and WT. RSV infection in SIRT1-deficient BMDC demonstrated a significantly increased level of pathological Th2 and Th17 cytokines IL-4, IL-5, IL-13 and IL-17. Furthermore, consistent with the decreased levels of *Il12*, RSV infection in SIRT1-deficient BMDC showed a decreased anti-viral IFN-γ (Fig 3F). Thus, it appears that mitochondrial dysfunction in DC is associated with altered innate immune cytokines that subsequently impact the activation of acquired immune response by inducing pathologic Th2 and Th17.

### Reverse Phase Protein Array (RPPA)

**Fig 4:**
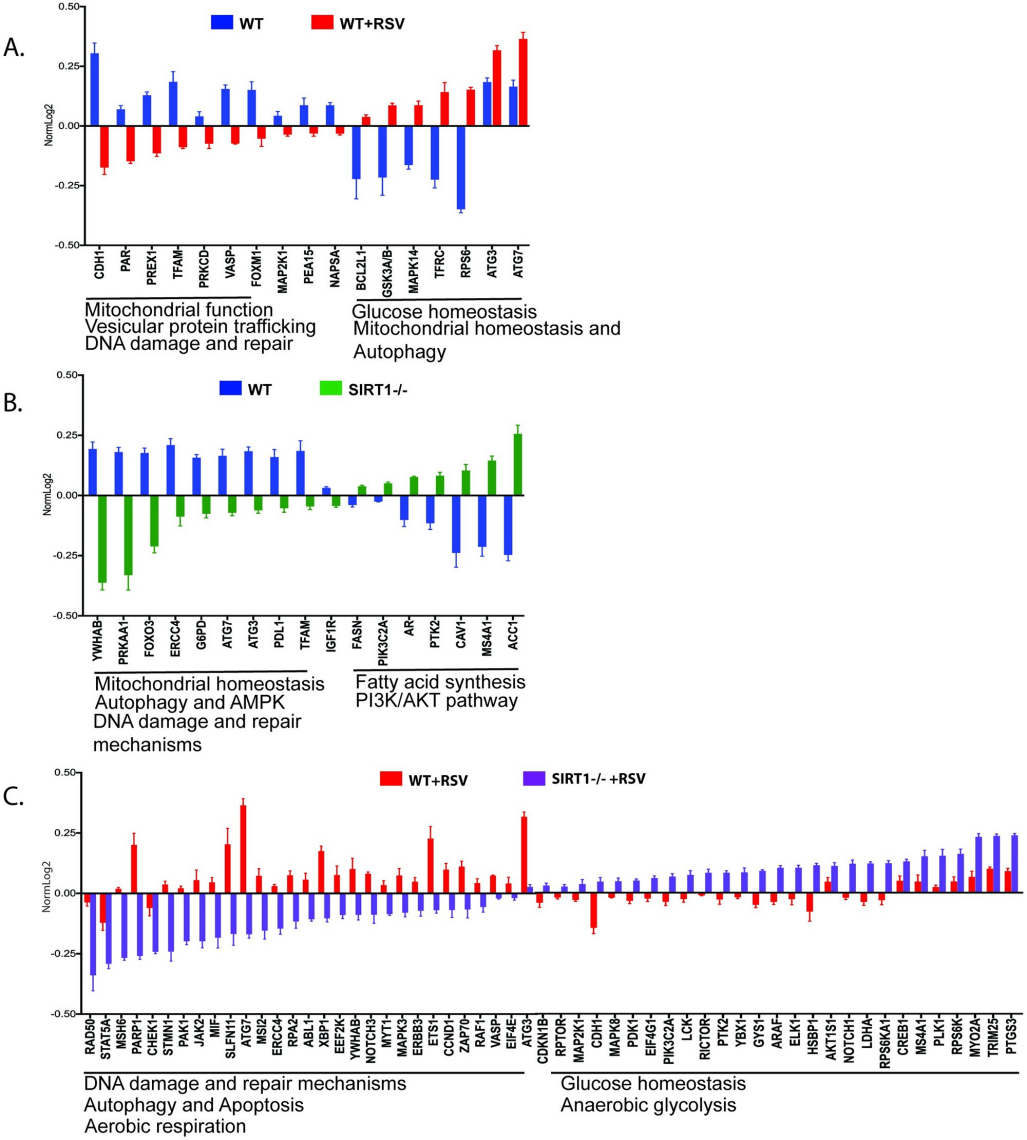
Reverse Phase Protein Array (RPPA) analysis. WT and SIRT1-deficient BMDC with or without RSV infection were subjected to RPPA analysis to identify differentially regulated proteins. (A) WT vs RSV infected WT BMDC (B) WT vs SIRT1-deficient BMDC without RSV infection (C) WT and SIRT1-deficient BMDC with RSV infection. RPPA is a commercially available facility at MD Anderson Cancer Centre, Texas, United States. *p* and *q* values determined using Graphpad prism7. Values represent Mean ± SEM. ***p< 0.0005 **p< 0.005 *p< 0.05.

Mitochondrial dysfunction leads to release of mitochondrial metabolites (including mtDNA and mitochondria-nucleus associated proteins) into the cytosol inducing oxidative and ER stress exacerbating inflammation [30]. Post-translational protein modifications play a critical role in modulating immune-metabolic signaling pathways. To explore if these processes are linked, a quantitative assessment of key proteins was analyzed in cell lysates of WT and SIRT1-/- BMDC using a proteomics based RPPA assessment. RPPA is an antibody-based proteomic technology suitable for profiling both total protein levels and posttranslational modifications, including phosphorylation, simultaneously across a large number of signaling pathways. All 3 comparisons (WT vs WT+RSV; WT vs SIRT1-/-; and WT+RSV vs SIRT1+RSV) identified a wide range of differentially regulated proteins that are closely related to the pathological innate and adaptive immune response pathways. RSV infection in WT BMDC compared to uninfected BMDC showed a reduced level of a set of key proteins that play a critical role in mitochondrial homeostasis, vesicular protein trafficking, and DNA repair mechanisms (Fig 4A; S1 Table). There were also increased levels of key proteins related to glucose and mitochondrial homeostasis as well as autophagy that may aid in reprogramming cellular metabolism linked to innate immune responses. The absence of SIRT1 in uninfected DC compared to WT DC resulted in the reduced levels of key metabolic proteins that are downstream of SIRT1, including AMPK pathway, oxidative pentose phosphate pathway (PPP), mitochondrial homeostasis, autophagy, and ER stress associated proteins. Absence of SIRT1 also resulted in increased levels of rate limiting enzymes of fatty acid synthesis, ACC1 (Acetyl CoA carboxylase alpha) and FASN (Fatty acid synthase) that are involved in de novo synthesis of fatty acids (Fig 4B; S2 Table). RSV infection in SIRT1-deficient BMDC compared to WT+RSV provoked reduced levels of a wide range of proteins that are primarily involved in oxidative and ER stress, DNA damage and repair mechanisms, apoptosis and autophagy (Fig 4C; S3 Table). Taken together, RPPA analyses demonstrate that RSV infection induced mitochondrial mediated oxidative and ER stress while the absence of SIRT1 impaired mitochondrial biogenesis and function that further resulted in the activation of fatty acid synthesis pathways.

### In vitro inhibition of ACC1 ameliorates mitochondrial stability and function

**Fig 5:**
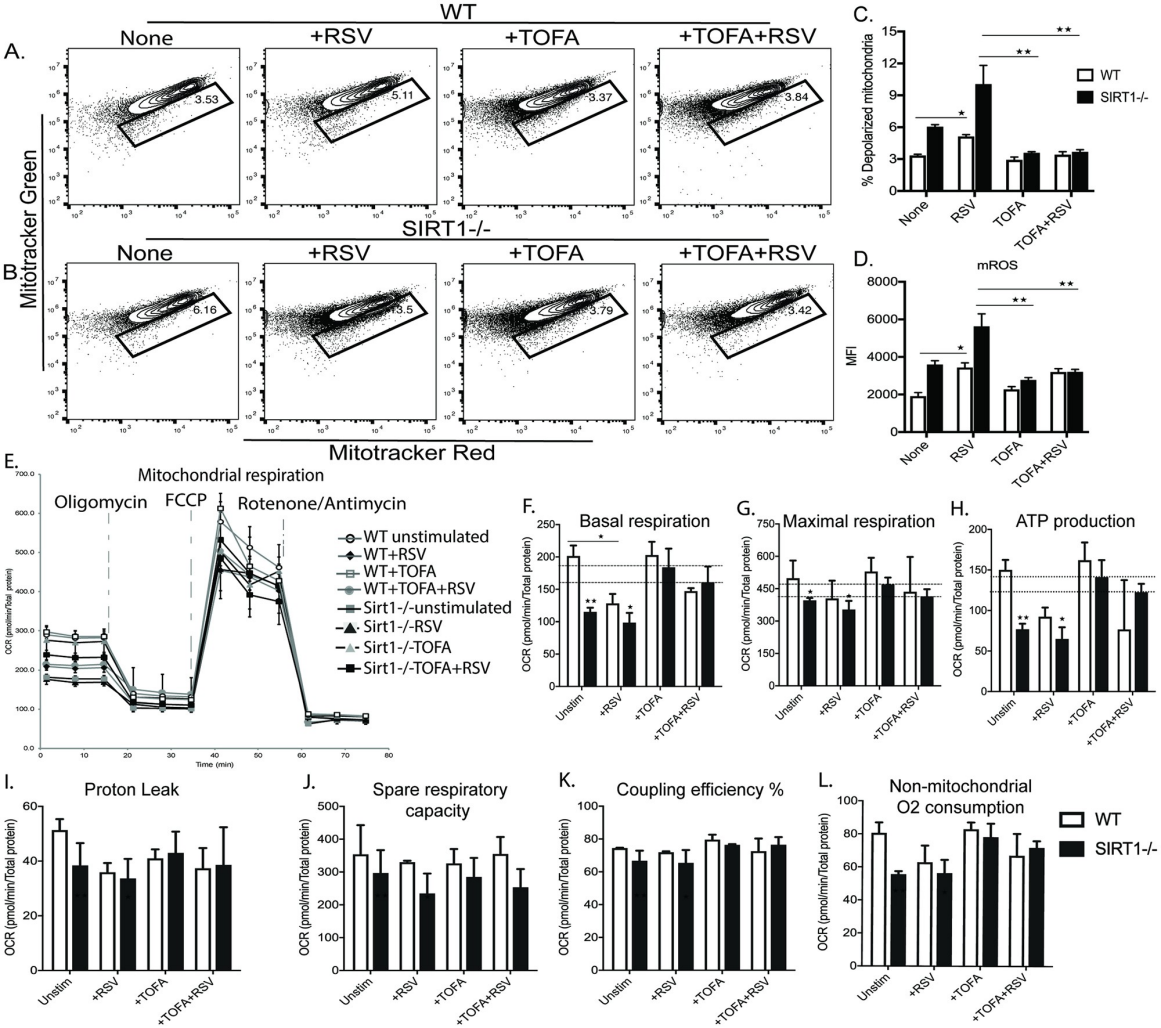
In vitro inhibition of ACC1 ameliorates mitochondrial membrane potential (Δ⍦*m*), and mitochondrial respiration. BMDC from WT C57BL/6 and SIRT1-deficient mice were cultured for 10 days in presence of GMCSF and treated with TOFA (5ug/mL) for 60min and then infected with RSV (MOI 2) for 2 hours. (A-B) Flow cytometry of WT and SIRT1-deficient BMDC with or without RSV infection using Mitogreen (50nM) and Mito Red (100nM). (C) Bar graph showing percent depolarized mitochondria. (D) Bar graph showing mROS determined using Mitosox (5uM) and analyzed with flow cytometer. (E-G) Representative OCR of WT and SIRT1-deficient BMDC with or without RSV infection incubated with TOFA. (H-L) Representative ATP production, Proton leak, Spare respiratory capacity; Coupling efficiency (%) and Non-mitochondrial oxygen consumption was generated using Seahorse XF Cell Mito stress report generator. Data are representative of at least 3 independent experiments with at least three replicates per group. Values represent Mean ± SD. ***p< 0.0005 **p< 0.005 *p< 0.05.

A central pathway that showed a remarkably increased activation in the absence of SIRT1 is catalyzed by an enzyme that regulates fatty acid synthesis, acetyl CoA carboxylase (ACC1). Since fatty acid synthesis and mitochondrial function have been implicated in regulation of various cytokines and mediators [32, 33], this pathway may represent a key in the metabolic reprogramming of DC and subsequent altered pathological immune responses. To investigate if blocking of ACC1 could ameliorate mitochondrial membrane potential and subsequent mROS generation, WT and SIRT1-deficient BMDC were treated with TOFA (5-(Tetradecyloxy)-2-furoic acid), a potent and specific inhibitor of ACC1, for 60 min prior to RSV infection and mitochondrial membrane potential was assessed using the same Mitotracker dyes as used in our earlier experiments. As observed earlier, RSV infection in WT BMDC demonstrated an increased number of depolarized mitochondria. However, TOFA treated WT BMDC infected with RSV (WT+TOFA+RSV) showed a decreased number of depolarized mitochondria (Fig 5A and 5B). Interestingly, TOFA treatment in SIRT1-deficient BMDC remarkably decreased the number of depolarized mitochondria even without RSV infection. This reinforces the notion that absence of SIRT1 induces baseline dysfunction to mitochondria (from Fig 2C), a phenotype rescued by ACC1 inhibition. Furthermore, TOFA treatment in RSV infected SIRT1-deficient BMDC (SIRT1+TOFA+RSV) has remarkably decreased the number of depolarized mitochondria not only compared to SIRT1+RSV but also as compared to uninfected SIRT1-deficient BMDC. It is apparent that the percentage of dysfunctional mitochondria in SIRT1+TOFA+RSV was similar to the uninfected WT BMDC (Fig 5A and 5B). Thus, ACC1 inhibition restored mitochondrial membrane potential in SIRT1-/- to WT BMDC levels. Amelioration of mitochondrial membrane depolarization by TOFA in the respective groups was also reflected in the relatively decreased mROS (Fig 5C). Thus, blocking of ACC1 ameliorates mitochondrial membrane potential alterations and mROS during RSV infection.

ACC1 inhibition was next tested for its effects on mitochondrial respiration. WT and SIRT1-deficient BMDC were treated with TOFA for 60 min prior to RSV infection, and oxygen consumption rate (OCR) was determined. As observed earlier, RSV infection in WT BMDC resulted in a decreased basal and maximal OCR as compared to uninfected WT BMDC. TOFA treated WT BMDC infected with RSV (WT+TOFA+RSV), however, did not show any change in OCR as compared to WT+RSV BMDC (Fig 5D–5F). Therefore, it appears that RSV mediated decreased mitochondrial respiration could occur through mitochondrial oxidative stress, but via activation of fatty acid synthesis. This is consistent with our RPPA data showing that RSV induced mitochondrial oxidative and/or ER stress did not affect any metabolic pathways. Interestingly, TOFA treated SIRT1-deficient BMDC (SIRT1+TOFA) showed 85% increase in basal OCR as compared to uninfected SIRT1-deficient BMDC. Similarly, TOFA treated SIRT1-deficient BMDC infected with RSV (SIRT1+TOFA+RSV) showed ~70% increased basal OCR as compared to SIRT1+RSV (Fig 5D–5F). A similar trend of OCR was apparent in maximal respiration and ATP production (Fig 5G–5K). However, TOFA treatment did not affect glycolytic function of any of the groups (S2 Fig). Thus, it is plausible that activated fatty acid synthesis in SIRT1-deficient BMDC disrupts mitochondrial function, and its inhibition with TOFA restored mitochondria to normal physiology that may reverse RSV induced immunopathology.

### In vitro inhibition of ACC1 regulates Innate and pathologic Th2 and Th17 immunity

**Fig 6:**
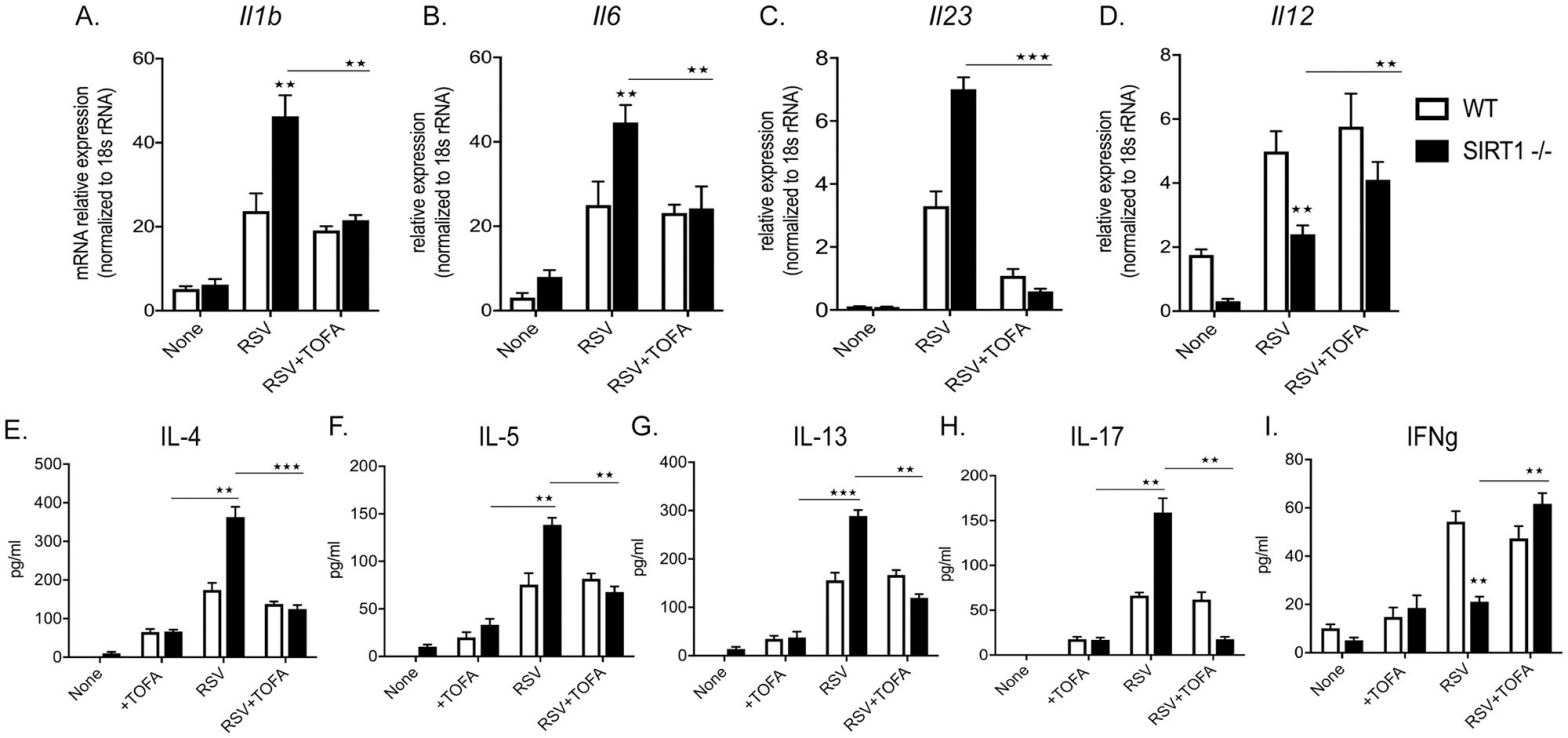
In vitro inhibition of ACC1 regulates Innate and pathologic Th2 and Th17 immunity. BMDC were grown from WT or Sirt^*fl/fl*^-CD11cCre+ mice. LDLNs from 8dpi RSV-infected WT mice were harvested and RSV responsive CD4+ T cells were isolated using Macs miltenyi CD4+ T cell isolation kit. Purified CD4+ T cells were then restimulated with WT or SIRT1-/- DC infected with RSV (MOI 2) overnight and supernatants collected after 48h. (A-D) mRNA expression of WT and SIRT1-deficient BMDC infected with RSV for 8h. (E-H) Cytokines secreted in 48h co-culture supernatants from DC and T cell co-cultures re-stimulated with RSV. Cultures were incubated with or without ACC1 inhibitor. Values are mean ± SEM. ***p< 0.0005 **p< 0.005 *p< 0.05.

Inhibition of fatty acid synthesis using synthetic inhibitors was shown to reduce RSV progeny replication [34], however, there are no reports on inhibition of fatty acid synthesis in the context of mitochondrial function and altered immune response in RSV infection. To investigate the impact of inhibition of fatty acid synthesis, WT and SIRT1-deficient BMDC were treated with TOFA for 60 min prior to RSV infection and gene expression analysis was done by qPCR. As observed earlier, RSV infection in SIRT1-deficient BMDC (SIRT1+RSV) induced a higher expression of *Il1β*, *Il6*, and *Il23* as compared to WT BMDC. But TOFA treatment in RSV infected SIRT1-deficient BMDC (SIRT1+TOFA+RSV) significantly decreased *Il1β* (54% decrease), *Il6* (46%), and *Il23* (92%) as compared to SIRT1+RSV (Fig 6A–6C). Interestingly, TOFA treatment in SIRT1+RSV BMDC induced 70% higher expression of *Il12* as compared to SIRT1+RSV (Fig 6D). Likewise, TOFA treatment in RSV infected SIRT1-deficient BMDC (SIRT1+TOFA+RSV) co-cultured with T cells from RSV infected animals resulted in significantly decreased levels of pathogenic cytokines IL-4 (65% decreased), IL-5 (51%), IL-13 (58%) and IL-17 (88%) as compared to RSV-infected SIRT1-/- BMDC (SIRT1+RSV) (Fig 6E–6G). Interestingly, TOFA treatment in SIRT1+RSV increased anti-viral IFN-γ by 190% as compared to SIRT1+RSV (Fig 6I). Thus, blocking of ACC1 restores mitochondrial and cellular metabolic function that mediates a more appropriate innate and adaptive RSV immune response.

### In vivo inhibition of fatty acid synthesis alleviates RSV induced lung pathology

**Fig 7:**
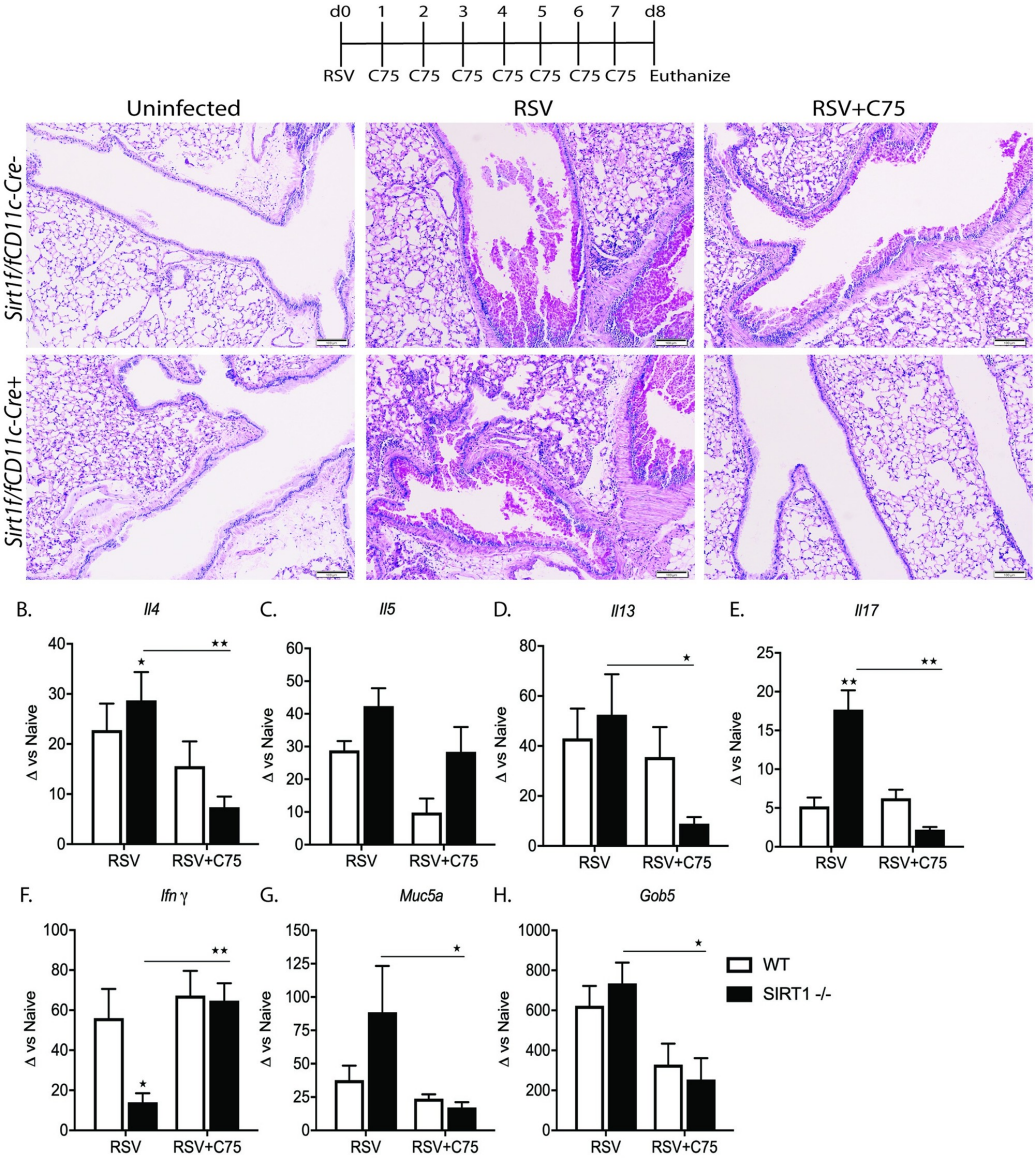
In vivo inhibition of fatty acid synthesis resolves RSV-induced exacerbated lung pathology. Animals were infected with RSV and treated daily with ACC1 inhibitor C75 as indicated. (A) Representative lung histology from naive and RSV infected *Sirt1*^*f/f*^*-CD11c-Cre* mice stained with hematoxylin. Scale bar, 100μm. (B-F) mRNA expression of lung cytokines, (G-H) mucus-associated gene Gob5 and Muc5ac were obtained using qPCR and compared with naive controls. Data representative of two independent experiments, 5 mice/group. Values are mean ± SEM. ***p< 0.0005 **p< 0.005 *p< 0.05.

The accumulation of free fatty acids induces inflammation and causes lipotoxic effects [35]. So, we hypothesized that the administration of C75, a potent fatty acid synthase inhibitor that is well tolerated *in vivo*, could alleviate the RSV-induced lung pathology. To test this hypothesis, mice were administered C75 daily to both WT and CD11c targeted SIRT1-/- mice infected with RSV. During the first 3d of RSV infection, the host response is dominated by innate immunity which includes the activation of resident DCs, the secretion of early inflammatory mediators, and the recruitment of NK cells, inflammatory DC and neutrophils [36]. As observed earlier in our previous publication [31], RSV-infected SIRT1-/- mice had greater increases in lung pathology and mucus production (Fig 7A). Importantly, C75 treatment in RSV-infected SIRT1-/- mice resolved the RSV-induced exacerbated lung pathology (Fig 7A, far right panel) showing remarkably reduced mucus, and reduced infiltration of immune cells. However, histological examination of the lungs from C75 treated RSV infected WT mice also revealed relatively reduced mucus and reduced inflammation (Fig 7A, upper far right panel). Consistent with the histopathology, SIRT1-/- mice had higher mRNA levels of potentially pathogenic cytokines *Il4*, *Il5*, *Il13*, and *Il17a* (Fig 7B–7E) as well as mucus-related genes *Muc5ac* and *Gob5* as compared to WT mice (Fig 7G–7H). Additionally, *Ifn*γ was downregulated in the lungs of SIRT1-/- mice post RSV infection (Fig 7F). However, C75 treatment in RSV-infected SIRT1-/- mice has significantly decreased pathogenic cytokines *Il4*, *Il13*, and *Il17a* (Fig 7B–7E), and mucus-related genes *Muc5ac* and *Gob5* as compared to RSV+SIRT1-/- mice treated with vehicle (Fig 7G and 7H). It is interesting to note that C75 treatment in SIRT1-/- mice showed a 4 four-fold increase in *Ifn*γ expression (Fig 7F), recapitulating our in vitro observations in BMDC/T cell coculture. Overall, these *in vivo* results parallel the *in vitro* results of ACC1 inhibitor studies and support the concept that SIRT1 mediated mitochondrial function regulates fatty acid synthesis leading to effective antiviral immunity and limits lung pathology.

## Discussion

**Fig 8:**
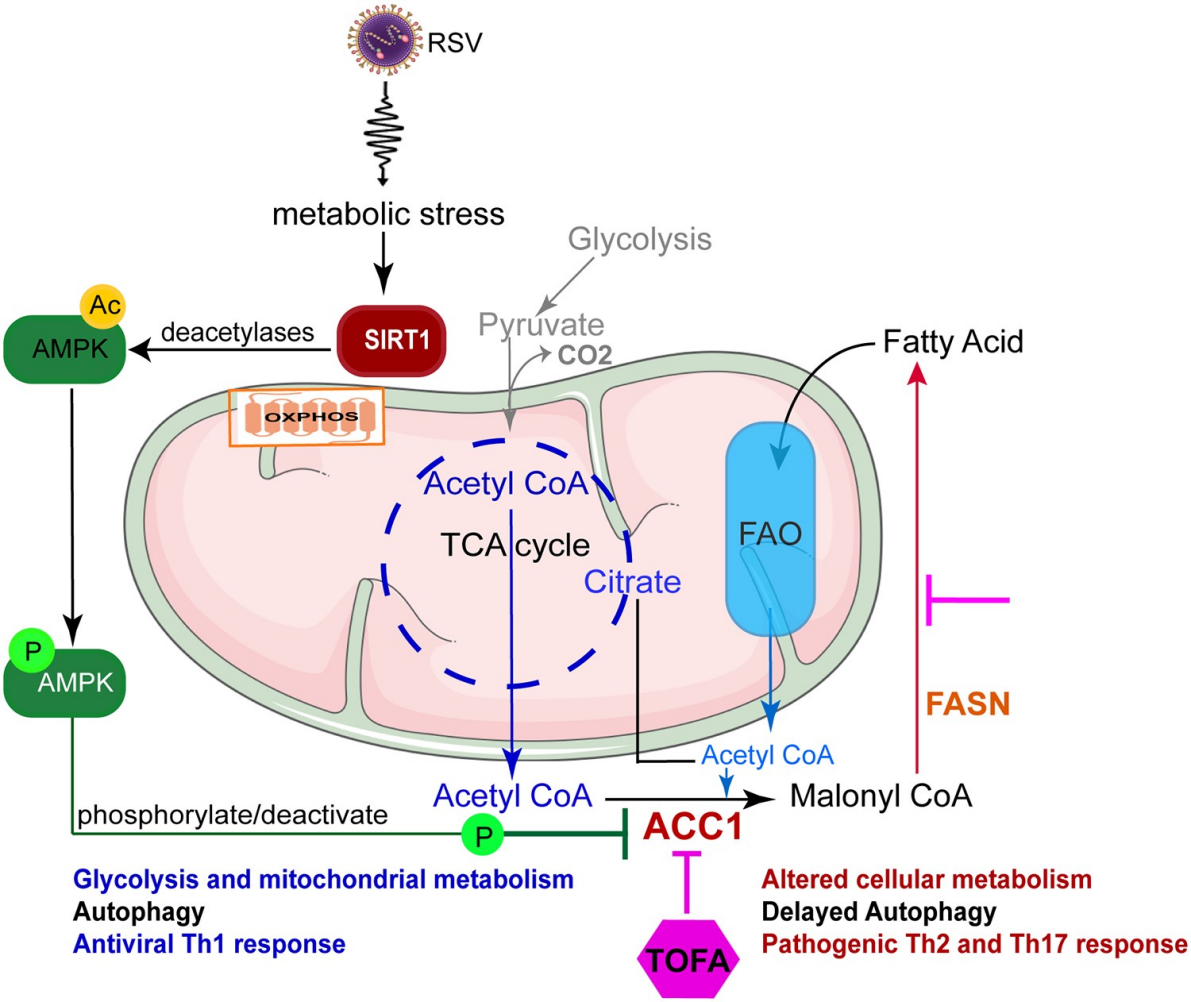
SIRT1 regulates fatty acid production leading to anti-viral innate immune response. During metabolic stress, SIRT1 translocate from nucleus to cytoplasm and activates the cellular energy sensing enzyme AMPK that stimulates catabolic processes such as glucose uptake and beta oxidation and decreases anabolic processes such as protein and fatty acid synthesis. AMPK then phosphorylate ACC1 and inactivates it so that the fatty acid synthesis is inhibited. ACC1 converts mitochondrial acetyl CoA to malonyl CoA (cytoplasmic), and fatty acid synthase (FASN) converts malonyl CoA to palmitate in multiple steps that enters the Fatty acid oxidation pathway in the mitochondria. Acetyl-coA pathway appears to centrally coordinate both mitochondrial function and cellular metabolism.

Dendritic cells are the primary antigen-presenting cells during RSV infection and play an important role in the initiation and phenotype of the immune response [37]. During infection DCs quickly switch from a resting to an activated state with a significant shift in metabolism to meet cellular bioenergetic and biosynthetic demands of the cells [38]. SIRT1 is involved in various physiological processes including aging, energy metabolism, and stress responses [39] and therefore has been considered a potential therapeutic target for multiple diseases like cancer, Alzheimer’s, diabetes, and atherosclerosis [40, 41]. These studies, and many others, have established that the immune response and metabolism are closely dependent on each other and related to SIRT1 regulation. Furthermore, the activation of SIRT1 by polyphenols, such as resveratrol and quercetin, have highlighted the connection of this pathway to health and diet. Mitochondria are the main organelles responsible for regulating cellular metabolism and have emerged as central in energy and immune homeostasis during ongoing disease responses. The mitochondrial machinery that includes mitochondrial DNA (mtDNA), metabolic pathways, mROS, mitophagy, antioxidant systems, and even mitochondrial dynamics are being extensively studied for their role in immune homeostasis [18]. In our current study we report that mitochondrial function regulates RSV-induced innate cytokine production leading to instruction of adaptive immune responses through SIRT1. The central role of ACC1 that activates Acetyl CoA was demonstrated in these studies, and as depicted in Fig 8, requires regulation by SIRT1 (via AMPK) in order to control the fatty acid synthesis pathway that leads to dysregulated innate cytokine responses. The inhibition of ACC1 in these studies allowed the SIRT1 deficient DC to manifest a more appropriate innate and acquired immune response. Thus, these studies establish several important paradigms that may be further considered for developing treatments during pathologic respiratory disease, including targeting Acetyl CoA activation.

RSV infection in WT BMDC induced dysfunctional mitochondria, characterized by increased levels of mROS indicate that RSV infection induces mitochondrial stress, directly or indirectly, by altering mitochondrial membrane potential and function. SIRT1 is important for mitochondrial metabolism, energy homeostasis and oxidative stress by regulating metabolic pathways including TCA cycle and OXPHOS [42] and dysfunctional mitochondria were further increased in SIRT1-/- DC. This was exemplified in the present studies where SIRT1-/- DC had significant changes in metabolic function and a broad shift in protein activation profiles that can alter innate cell function. Mitochondrial stress in SIRT1 BMDC was evident with reduced basal and maximal respiration rate that also reflected in the decreased ATP production. Surprisingly, RSV infection in SIRT1 BMDC did not induce further reduction in basal and maximal respiration rate, likely due to the dominant effect of absence of SIRT1, to the cumulative effect of increased mROS, and/or to compensation by non-mitochondrial energy production via glycolysis [43, 44]. Our data clearly indicate that OXPHOS in SIRT1-deficient cells was impaired due to dysfunctional mitochondria, associated with generated excess ROS production leading to DNA damage and oxidative stress. ROS, at low concentrations, regulates homeostatic signaling cascades, while at higher concentrations oxidizes DNA, lipids and crucial signaling cellular proteins leading to cellular damage and inflammation [45]. ROS formation is a constant process in every cell, primarily due to OXPHOS. Increased ROS levels can also be generated via increased stimulation of NADPH oxidase (mitochondrial and cell membrane associated protein), or in the contexts of mitochondrial dysfunction or impaired activity of antioxidant pathways [45]. RSV infection in airway epithelial cells induce excess ROS production, leading to cellular oxidative damage [46], and inducing an imbalance between ROS production and antioxidant cellular defenses [47, 48]. Antioxidant treatment has been shown to ameliorate RSV-induced disease and lung inflammation [49]. Thus, the overall effect of SIRT1 deficiency was the loss of the ability to respond appropriately to cellular and mitochondrial metabolic stress.

Innate immunity is the first line of host defense through direct engagement of pathogens that subsequently initiates the development of an adaptive immune response. Recent literature has substantiated the importance of mitochondrial membrane integrity in innate immune activation [23, 27]. RSV infection in WT and SIRT1 BMDC significantly induced innate cytokine genes *Il1β*, *ll6*, and *Il23*, but lack of SIRT1 was associated with a more pronounced cytokine expression. However, lack of SIRT1 inhibited *Il12*, which is central for inducing IFNγ as a primary anti-RSV immune response. Induced *Il1β*, *ll6*, and *Il23* cytokines in SIRT1 DC correlated to the remarkably elevated levels of IL-17 secretion from lymph node restimulated cells. IL-17 mediates mucus hypersecretion, airway hypersensitivity and granulocytic chemotaxis in the inflamed lungs [50, 51] and these same profile of cytokines have been associated with severe disease in infants. Lack of SIRT1 also induced elevated levels of IL-4, IL-5 and IL-13, and together with IL-17, led to heightened pathologic responses within the airways (15) and consistent with previous reports on SIRT1 from our laboratory [31]. Increased levels of IL-1b, IL-6, IL-4 and IL-13 also upregulates the expression and production of airway mucins in airway epithelial cells [52]. In related responses, mitochondrial dysfunction was reported in mouse models of allergic asthma, and was related to the severity of ongoing responses [53–55]. Elevated levels of IL-4 together with ADMA (asymmetric dimethyl arginine) were shown to potently induce mitochondrial dysfunction, increased mROS and reduced mitochondrial mass in airway epithelial cells [56].

Mitochondrial dysfunction, oxidative stress, and ER stress are intricately linked processes. Mitochondria generate ATP by utilizing the substrates from glucose and lipid metabolism, and when mitochondrial oxygen consumption is hampered due to an altered metabolism, excess ROS are generated that induce oxidation causing damage to DNA, proteins and lipids [57, 58]. All of these interlinked processes are part of normal physiology; however, alterations are linked to the pathophysiology of several diseases involving acute and chronic inflammation [59]. RPPA analysis of RSV infected DC revealed a range of differentially regulated proteins that are important for mitochondrial function, glucose homeostasis, autophagy, vesicular transport protein process, and DNA repair. RSV infection destabilized mitochondria and decreased OXPHOS in DC were evident in the Seahorse respirometry analyses. Despite the mitochondrial stress, glucose homeostasis was highly upregulated to support cell survival and activation that contributes to altered immune responses.

RPPA analysis of SIRT1-deficient BMDC revealed changes in a number of key proteins linked to metabolic pathways downstream of SIRT1 including fatty acid synthesis, oxidative pentose-phosphate, and, not surprisingly, AMPK. The central molecule that is integral for the TCA/Kreb’s cycle, fatty acid synthesis and beta-oxidation is acetyl-CoA [60]. AMPK phosphorylates the rate-limiting enzyme of fatty acid synthesis, ACC1, and inactivates it so that malonyl CoA is not formed and the fatty acid synthesis is inhibited [61, 62]. This process is defective in SIRT1-deficient DC. Thus, the over activated acetyl-coA pathway appears to centrally coordinate both mitochondrial function and cellular metabolism. Inhibition of ACC1 blocks fatty acid synthesis in its entirety, including FASN (Fatty acid synthase), so it is likely that the resultant metabolic and immune phenotype is due to the cumulative effect of inhibition of ACC1 and FASN. The inhibition of ACC1 with a specific inhibitor led to correction of the altered metabolic state and resulted in the stabilization of the altered innate and acquired immune responses driven by RSV in DC and altered the pathologic responses in the lung. By identifying key metabolic pathways and demonstrating that we can interrupt the detrimental immune phenotypes by regulating their function, new therapeutics for RSV may emerge that either already exist or can be developed.

## Materials and methods

### Ethics statement

All work involving animals was reviewed and approved by the University of Michigan University Committee on Care and Use of Animals (Animal welfare assurance #A3114-01). The University of Michigan is an AAALAC international accredited animal care organization and all animals are housed in secure SPF conditions and follows the Office of Laboratory Animal Welfare (OLAW) guidelines as well as the US Department of Agriculture Animal and Plant Health Inspection Service guidelines.

### Reagents

Acetyl CoA carboxylase inhibitor, TOFA (5-(tetradecyloxy)-2-furancarboxylic acid) (Cayman, Ann Arbor, USA) was reconstituted in Ethanol and diluted in culture medium for in vitro work. We verified 5μM as an appropriate dose in vitro [63], with no significant changes in DC cytokine production at greater concentrations. BMDC were treated with TOFA for 60min prior to RSV infection. We observed comparable viability in control and TOFA-treated cells by exclusion dye stain (trypan blue). In the in vivo experiments, RSV infected mice received daily i.p. injections of 100μl C75 (4-Methylene-2-octyl-5-oxotetrahydrofuran-3-carboxylic acid) 5mg/kg [35] reconstituted in DMSO and diluted in normal saline; control animals received DMSO-saline.

### Mice

C57BL/6J (BL6), B6;129-*Sirt1*^*tm1Ygu*^*/J*(*Sirt1*^*f/f*^), and C57BL/6J-Tg (Itgax-Cre-EGFP) 4097Ach/J (CD11c-Cre-GFP) mice were purchased at 6–7 weeks of age from the Jackson Laboratory (Bar Harbor, ME). *Sirt1*^*f/f*^ mice, in which two loxP sites flank *Sirt1* exon 4, were crossed to CD11c-Cre-GFP transgenic mice. As the *Sirt1*^*f/f*^ mice were on a mixed C57BL/6J;129 background, we backcrossed the *Sirt1*^*f/f*^*-CD11c-Cre* progeny to a C57BL/6J background for six generations. Deletion of exon 4 produces an internally truncated protein that lacks catalytic activity, causing a Sirt1-null genotype [64]. Thus, Cre+ mice lack a functional SIRT1 in CD11c^high^ cells. *Sirt1*^*f/f*^-*CD11c-Cre* (SIRT1-/-) mouse breeding took place in-house at the University of Michigan (Ann Arbor, MI).

### RSV and plaque assays

Line 19 RSV (antigenic subgroup A), originally obtained from a sick infant at the University of Michigan Hospital System, was shown in animal models to mimic human infection by eliciting airway mucus production upon inoculation with 1×10^5^ PFU RSV. RSV strains were propagated in our laboratory in HEp-2 cells (American Type Culture Collection). Mice were infected intratracheally with 1×10^5^ PFU RSV as previously described [65]. Plaque assays were performed on RSV-infected lungs. Whole lungs were harvested 4d post infection (dpi) and ground with sand using a mortar and pestle. Supernatants were serially diluted and incubated with Vero cells for 3d. RSV plaques were detected using a specific polyclonal Ab (Millipore, Temecula, CA).

### BMDC culture

Bone marrow-derived dendritic cells (BMDC) were isolated from whole bone marrow of C57BL/6J wild type littermates (WT) and *Sirt1*^*f/f*^ CD11c-Cre (SIRT1-/-). Bone marrow cells were seeded into tissue culture flasks containing RPMI 1640 complete media supplemented with 20 ng GM-CSF/ml (R&D Systems, Minneapolis, USA). BMDC were fed on days 3, 5, 7, 9 and harvested on day 10, a time point by which cells were ≥ 85% CD11b+ CD11c+ DC by flow cytometric analysis. Cells derived from the *Sirt1*^*f/f*^*-CD11c-Cre* mice were cultured for 10 d (fed on days 3, 5, and 7, 9) to achieve high *Cre* activity. BMDC were infected with RSV (multiplicity of infection; MOI 2) for 2 hours and washed 3 times with RPMI 1640 complete media for specific assays and treatments.

### Reverse Phase Protein Array (RPPA)

BMDC lysate was prepared according to the instructions of the RPPA core facility, MD Anderson Cancer Center, Texas, USA. Briefly, 0.5x10^6^ BMDC per 3mL RPMI complete media in each well of a 6 well plate was infected with, and without RSV (MOI 2) for 2 hours. Cells were then washed with PBS three times before adding lysis buffer to each well (cat # 9803, Cell Signaling, USA), and followed the manufacturer’s instructions to collect the supernatant for further use. Protease and Phosphatase inhibitor cocktail, EDTA-free (cat # 78441 Thermo scientific) was added to the supernatant and sent to RPPA facility for processing and analysis. Data generated was analyzed using gene ontology (GO) tools; and *q* values were determined using the Two-stage linear step-up procedure of Benjamini, Krieger and Yekutieli, with Q = 10% using Graphpad Prism 7.

### Flow cytometry

BMDC cultured for 10 days were infected with or without RSV were stained with Mitotracker Green FM (50nM) to measure mitochondrial mass (cat# M7514, Thermo Fisher, USA), and Mitotracker Red FM (100nM) (cat# M22425, Thermo Fisher, USA) to detect mitochondrial membrane depolarization, in PBS for 30min in dark and on ice, according to the manufacturer’s instructions. Mitotracker Green FM stains all the mitochondria irrespective of its membrane depolarization while the Mitotracker Red FM stains only the depolarized mitochondria. In a separate tube, BMDC were stained with mitochondrial specific reactive oxygen species (mROS) dye, Mitosox (5μM) in PBS for 30min in dark and on ice. Cells were then washed 3 times with PBS and analyzed using Novocyte flow cytometer. Data analysis was performed using the FlowJo software (TreeStar, Ashland, OR).

### Seahorse assay

The Seahorse XFe96 Analyzer (Agilent Technologies, Santa Clara, CA, USA) was used to measure the mitochondrial function of BMDC. Briefly, 1x10^5^ cells per well, cultured in RPMI complete media supplemented with 10% (v/v) heat-inactivated FBS were plated in a 96 well Seahorse plate. BMDC were infected with RSV for 2h, then washed twice with Seahorse assay media (Agilent Technologies, Santa Clara, CA, USA) and incubated for 30 min in a CO_2_ free incubator at 37°C. In specific experiments, BMDC were treated with TOFA for 60min before RSV infection. Oxygen consumption rate (OCR) was determined using a cell Mito Stress Test kit (Agilent Technologies, Santa Clara, CA, USA) according to manufacturer’s instructions. Oligomycin (2μM); FCCP (Carbonyl cyanide 4-(trifluoromethoxy) phenylhydrazone) (1.5μM); Rotenone (0.5μM), and Antimycin (0.5μM) were used in the assay. Glycolytic function was determined using Glyco stress test kit (Agilent Technologies, Santa Clara, CA, USA). Glucose free media was used in the determination of Glycolytic function. Glucose (10mM); Oligomycin (2μM); and 2-deoxy D-glucose (2-DG) (50mM) were used in the glycolysis assay. After the assay cell lysate was made using Ripa cell lysis and extraction buffer (Thermo scientific) and protein was estimated using BCA protein assay kit (Pierce Thermo scientific); protein values were used to normalize OCR and ECAR values as recommended by the manufacturer using Wave 2.0 desktop software. Normalized data was further analyzed for interpretation using Seahorse XF Cell Mito stress test Report generator and Seahorse XF Cell Glycolysis stress test Report generator. Data generated in Seahorse Report generator (Mean ± SD) was extrapolated in Graphpad Prism 7.0.

### Lymph node restimulation

For DC-T cell co-culture experiments, RSV-responsive CD4+ T cells were obtained from mediastinal lymph nodes (MLN) of RSV-infected mice, 8 days post-infection. Cells were purified using a magnetic column negative selection protocol to isolate CD4+ T cells (Miltenyi Biotech, 98% pure; isolation kit #130104454). For RSV-reactive T cell co-cultures, DCs were infected with RSV MOI of 2 hours. The DC were then washed and placed into culture with isolated T cells from lung draining lymph nodes of RSV infected mice at a 1:10 ratio of DC to T cells. Culture supernatants were harvested at 48 hours post-co-culture and analyzed using a customized Bioplex assay (Biorad).

### Quantitative PCR

Total RNA was isolated from BMDC culture or lung lobe using TRIzol reagent, according to manufacturer’s instructions (Invitrogen, Grand Island, NY). RNA was reverse transcribed, and cytokine gene expression was assessed using TaqMan Gene Expression Assay primer/probe sets on an ABI Prism 7500 Sequence Detection System (Applied Biosystems, Foster City, CA). Custom primers were used to assess transcription levels of RSV-G, RSV-F, Muc5a and Gob5, as previously described [66]. Fold change expression was calculated from gene expression values normalized to 18s RNA and or naïve animals.

### Lung histology

Serial 6-μm sections were obtained from paraffin-embedded, 10% formalin-fixed left lungs stained with H&E. Five sections were analyzed per mouse lung, with two lung slices per section per mouse to select representative slides.

### Statistical analysis

Data were analyzed and graphed using the GraphPad Prism 7 software (San Diego, CA). Statistical significance was determined by Two way ANOVA and Bonferroni post-t-test to obtain *p*-values. Adjusted *p*-values below 0.05 are considered statistically significant.

## Supporting information

S1 Fig Absence of SIRT1 in BMDC induced glycolysis. BMDC from WT C57BL/6 and SIRT1-deficient mice were cultured for 10 days in presence of GMCSF and infected with RSV MOI 2 for 2 hours. Glycolytic function was determined using Glyco stress test kit and analyzed with Seahorse XFe96 analyzer. (A-B) representative ECAR of WT and SIRT1-deficient BMDC with or without RSV infection. (C-E) Representative Glycolytic capacity, Glycolytic Reserve and Non-Glycolytic Acidification was generated using Seahorse XF Cell Glyco stress report generator. Data is representative of 3 independent experiments with three replicates per group. Values represent Mean+SD. *p< 0.05.
(TIF)

S2 Fig TOFA induced glycolysis in SIRT1-deficient BMDC during RSV infection. BMDC from WT C57BL/6 and SIRT1-deficient mice were cultured for 10days in presence of GMCSF. BMDC were incubated with TOFA for 60min prior to RSV infection. Glycolytic function was determined using Glyco stress test kit and analyzed with Seahorse XFe96 analyzer. (A-B) Representative ECAR of WT and SIRT1-deficient BMDC with or without RSV infection. (C-E) Representative Glycolytic capacity, Glycolytic Reserve and Non-Glycolytic Acidification was generated using Seahorse XF Cell Glyco stress report generator. Data are representative of at least 3 independent experiments with at least three replicates per group. Values represent Mean ± SD. *p< 0.05.
(TIF)

S1 Table Differentially regulated proteins in WT BMDC infected with respiratory syncytial virus (RSV) compared to uninfected WT BMDC. (DOCX)

S2 Table Differentially regulated proteins in SIRT1-deficient (SIRT1-/-) BMDC compared to WT BMDC. (DOCX)

S3 Table Differentially regulated proteins in SIRT1-deficient (SIRT1-/-) BMDC infected with respiratory syncytial virus (RSV) compared to WT BMDC. (DOCX)
