# Supplementary figures and images for: Sirtuin 1 regulates mitochondrial function and immune homeostasis in respiratory syncytial virus infected dendritic cells
Source: PLoS Pathog. 2020 Feb 27;16(2):e1008319. doi: 10.1371/journal.ppat.1008319 (PMC7046194; doi:10.1371/journal.ppat.1008319)

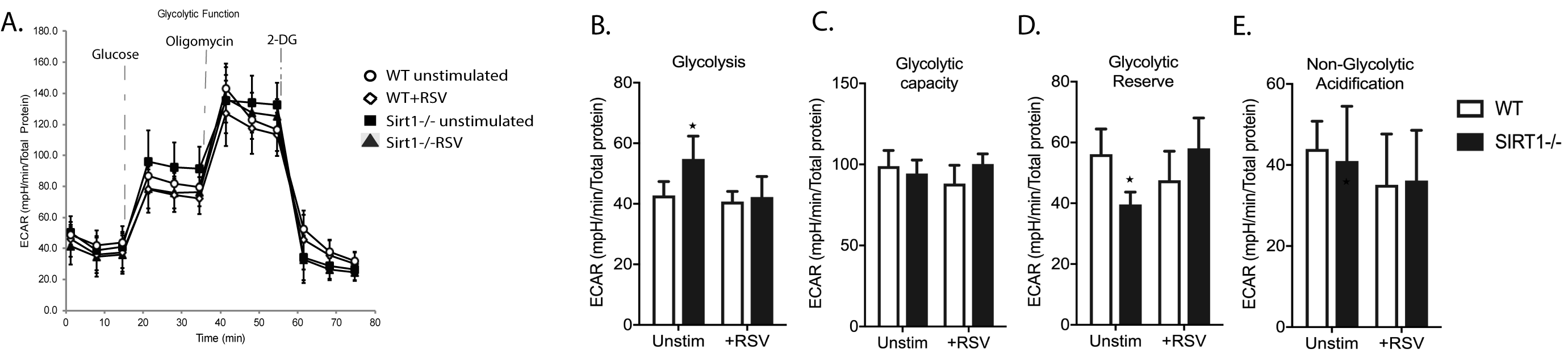

Supplement: S1 Fig — BMDC from WT C57BL/6 and SIRT1-deficient mice were cultured for 10 days in presence of GMCSF and infected with RSV MOI 2 for 2 hours. Glycolytic function was determined using Glyco stress test kit and analyzed with Seahorse XFe96 analyzer. (A-B) representative ECAR of WT and SIRT1-deficient BMDC with or without RSV infection. (C-E) Representative Glycolytic capacity, Glycolytic Reserve and Non-Glycolytic Acidification was generated using Seahorse XF Cell Glyco stress report generator. Data is representative of 3 independent experiments with three replicates per group. Values represent Mean+SD. *p< 0.05. (TIF) [file ppat.1008319.s001.tif]

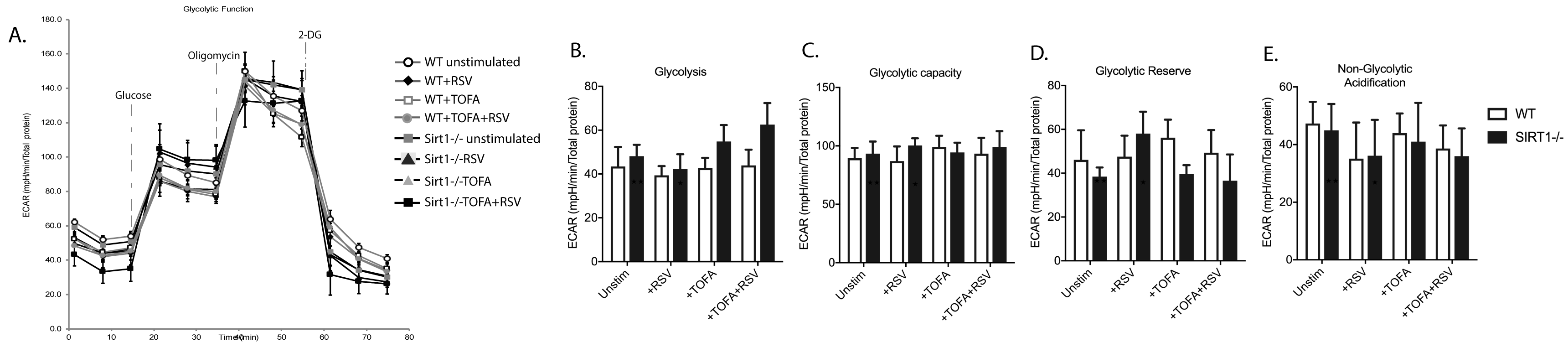

Supplement: S2 Fig — BMDC from WT C57BL/6 and SIRT1-deficient mice were cultured for 10days in presence of GMCSF. BMDC were incubated with TOFA for 60min prior to RSV infection. Glycolytic function was determined using Glyco stress test kit and analyzed with Seahorse XFe96 analyzer. (A-B) Representative ECAR of WT and SIRT1-deficient BMDC with or without RSV infection. (C-E) Representative Glycolytic capacity, Glycolytic Reserve and Non-Glycolytic Acidification was generated using Seahorse XF Cell Glyco stress report generator. Data are representative of at least 3 independent experiments with at least three replicates per group. Values represent Mean ± SD. *p< 0.05. (TIF) [file ppat.1008319.s002.tif]
