# Supplementary material for: Sirtuin 1 regulates mitochondrial function and immune homeostasis in respiratory syncytial virus infected dendritic cells
Source: PLoS Pathog. 2020 Feb 27;16(2):e1008319. doi: 10.1371/journal.ppat.1008319 (PMC7046194; doi:10.1371/journal.ppat.1008319)
Supplement: S1 Table — (DOCX) [file ppat.1008319.s003.docx]

S1 Table. Differentially regulated proteins in WT BMDC infected with respiratory syncytial virus (RSV) compared to Uninfected WT BMDC

|  | Gene | UniProtKB accession # | Description |
| --- | --- | --- | --- |
| 1 | CDH1 | [P12830](http://www.uniprot.org/entry/P12830) | Cadherin 1 |
| 2 | PAR | [Q8TEW0](http://www.uniprot.org/entry/Q8TEW0) | Partitioning defective 3 homolog |
| 3 | PREX1 | [Q8TCU6](http://www.uniprot.org/entry/Q8TCU6) | Phosphatidylinositol-3,4,5-trisphosphate dependent Rac exchange factor 1 |
| 4 | TFAM | [Q00059](http://www.uniprot.org/entry/Q00059) | Transcription factor A, mitochondrial |
| 5 | PRKCD | [Q05655](http://www.uniprot.org/entry/Q05655) | Protein kinase C delta |
| 6 | VASP | [P50552](http://www.uniprot.org/entry/P50552) | Vasodilator-stimulated phosphoprotein |
| 7 | FOXM1 | [Q08050](http://www.uniprot.org/entry/Q08050) | Forkhead box M1 |
| 8 | MAP2K1 | [Q02750](http://www.uniprot.org/entry/Q02750) | Mitogen-activated protein kinase kinase 1 |
| 9 | PEA15 | [Q15121](http://www.uniprot.org/entry/Q15121) | Phosphoprotein enriched in astrocytes 15 |
| 10 | NAPSA | [O96009](http://www.uniprot.org/entry/O96009) | Napsin A aspartic peptidase |
| 11 | BCL2L1 | [Q07817](http://www.uniprot.org/entry/Q07817) | BCL2 like 1 |
| 12 | GSK3A/ GSK3B | [P49840](http://www.uniprot.org/entry/P49840) | Glycogen synthase kinase 3 alpha |
| 13 | MAPK14 | [Q16539](http://www.uniprot.org/entry/Q16539) | Mitogen-activated protein kinase 14 |
| 14 | RPS6 | [P62753](http://www.uniprot.org/entry/P62753) | Ribosomal protein S6 |
| 15 | TFRC | [P02786](http://www.uniprot.org/entry/P02786) | Transferrin receptor |
