# Supplementary material for: Sirtuin 1 regulates mitochondrial function and immune homeostasis in respiratory syncytial virus infected dendritic cells
Source: PLoS Pathog. 2020 Feb 27;16(2):e1008319. doi: 10.1371/journal.ppat.1008319 (PMC7046194; doi:10.1371/journal.ppat.1008319)
Supplement: S2 Table — (DOCX) [file ppat.1008319.s004.docx]

S2 Table. Differentially regulated proteins in SIRT1-deficient (SIRT1-/-) BMDC compared to WT BMDC

|  | Gene | UniProtKB accession # | Description |
| --- | --- | --- | --- |
| 1 | YWHAB | [P31946](http://www.uniprot.org/entry/P31946) | Tyrosine 3-monooxygenase/tryptophan 5-monooxygenase activation protein beta |
| 2 | PRKAA1 | [Q13131](http://www.uniprot.org/entry/Q13131) | Protein kinase AMP-activated catalytic subunit alpha 1 |
| 3 | FOXO3 | [O43524](http://www.uniprot.org/entry/O43524) | Forkhead box O3 |
| 4 | ERCC4 | [Q92889](http://www.uniprot.org/entry/Q92889) | ERCC excision repair 4, endonuclease catalytic subunit |
| 5 | ATG3 | [Q9NT62](http://www.uniprot.org/entry/Q9NT62) | Autophagy related 3 |
| 6 | ATG7 | [O95352](http://www.uniprot.org/entry/O95352) | Autophagy related 7 |
| 7 | CD274/PDL1 | [Q9NZQ7](http://www.uniprot.org/entry/Q9NZQ7) | CD274 molecule |
| 8 | G6PD | [P11413](http://www.uniprot.org/entry/P11413) | Glucose-6-phosphate dehydrogenase |
| 9 | IGF1R | [P08069](http://www.uniprot.org/entry/P08069) | Insulin like growth factor 1 receptor |
| 10 | FASN | [P49327](http://www.uniprot.org/entry/P49327) | Fatty acid synthase |
| 11 | PIK3C2A | [O00443](http://www.uniprot.org/entry/O00443) | Phosphatidylinositol-4-phosphate 3-kinase catalytic subunit type 2 alpha |
| 12 | AR | [P10275](http://www.uniprot.org/entry/P10275) | Androgen receptor |
| 13 | PTK2 | [Q05397](http://www.uniprot.org/entry/Q05397) | Protein tyrosine kinase 2 |
| 14 | CAV1 | [Q03135](http://www.uniprot.org/entry/Q03135) | Caveolin 1 |
| 15 | MS4A1 | [P11836](http://www.uniprot.org/entry/P11836) | Membrane spanning 4-domains A1 |
| 16 | ACACA | [Q13085](http://www.uniprot.org/entry/Q13085) | Acetyl-CoA carboxylase alpha |
