# Supplementary material for: Sirtuin 1 regulates mitochondrial function and immune homeostasis in respiratory syncytial virus infected dendritic cells
Source: PLoS Pathog. 2020 Feb 27;16(2):e1008319. doi: 10.1371/journal.ppat.1008319 (PMC7046194; doi:10.1371/journal.ppat.1008319)
Supplement: S3 Table — (DOCX) [file ppat.1008319.s005.docx]

S3 Table. Differentially regulated proteins in SIRT1-deficient (SIRT1-/-) BMDC infected with respiratory syncytial virus (RSV) compared to WT BMDC

| Gene | UniProtKB accession # | Description |
| --- | --- | --- |
| RAD50 | [Q92878](http://www.uniprot.org/entry/Q92878) | RAD50 double strand break repair protein |
| STAT5A | [P42229](http://www.uniprot.org/entry/P42229) | Signal transducer and activator of transcription 5A |
| MSH6 | [P52701](http://www.uniprot.org/entry/P52701) | MutS homolog 6 |
| PARP1 | [P09874](http://www.uniprot.org/entry/P09874) | Poly(ADP-ribose) polymerase 1 |
| CHEK1 | [O14757](http://www.uniprot.org/entry/O14757) | Checkpoint kinase 1 |
| STMN1 | [P16949](http://www.uniprot.org/entry/P16949) | Stathmin 1 |
| PAK1 | [Q13153](http://www.uniprot.org/entry/Q13153) | P21 (RAC1) activated kinase 1 |
| JAK2 | [O60674](http://www.uniprot.org/entry/O60674) | Janus kinase 2 |
| MIF | [P14174](http://www.uniprot.org/entry/P14174) | Macrophage migration inhibitory factor (glycosylation-inhibiting factor) |
| SLFN11 | [Q7Z7L1](http://www.uniprot.org/entry/Q7Z7L1) | Schlafen family member 11 |
| MSI2 | [Q96DH6](http://www.uniprot.org/entry/Q96DH6) | Musashi RNA binding protein 2 |
| ERCC4 | [Q92889](http://www.uniprot.org/entry/Q92889) | ERCC excision repair 4, endonuclease catalytic subunit (HGNC Symbol) |
| RPA2 | [P15927](http://www.uniprot.org/entry/P15927) | Replication protein A2 |
| ABL1 | [P00519](http://www.uniprot.org/entry/P00519) | ABL proto-oncogene 1, non-receptor tyrosine kinase |
| XBP1 | [P17861](http://www.uniprot.org/entry/P17861) | X-box binding protein 1 |
| TSC2 | [P49815](http://www.uniprot.org/entry/P49815) | Tuberous sclerosis 2 |
| EEF2K | [O00418](http://www.uniprot.org/entry/O00418) | Eukaryotic elongation factor 2 kinase |
| YWHAB | P31946 | Tyrosine 3-monooxygenase/tryptophan 5-monooxygenase activation protein beta |
| NOTCH3 | [Q9UM47](http://www.uniprot.org/entry/Q9UM47) | notch 3 |
| MYT1 | [Q01538](http://www.uniprot.org/entry/Q01538) | Myelin transcription factor 1 |
| MAPK3 | [P27361](http://www.uniprot.org/entry/P27361) | Mitogen-activated protein kinase 3 |
| ERBB3 | [P21860](http://www.uniprot.org/entry/P21860) | Erb-b2 receptor tyrosine kinase 3 |
| ETS1 | [P14921](http://www.uniprot.org/entry/P14921) | ETS proto-oncogene 1, transcription factor |
| CCND1 | [P24385](http://www.uniprot.org/entry/P24385) | Cyclin D1 |
| ZAP70 | [P43403](http://www.uniprot.org/entry/P43403) | Zeta chain of T-cell receptor associated protein kinase 70 |
| RAF1 | [P04049](http://www.uniprot.org/entry/P04049) | Raf-1 proto-oncogene, serine/threonine kinase |
| VASP | [P50552](http://www.uniprot.org/entry/P50552) | Vasodilator-stimulated phosphoprotein |
| EIF4E | [P06730](http://www.uniprot.org/entry/P06730) | Eukaryotic translation initiation factor 4E |
| CDKN1B | [P46527](http://www.uniprot.org/entry/P46527) | Cyclin dependent kinase inhibitor 1B |
| RPTOR | [Q8N122](http://www.uniprot.org/entry/Q8N122) | Regulatory associated protein of MTOR complex 1 |
| MAP2K1 | [Q02750](http://www.uniprot.org/entry/Q02750) | Mitogen-activated protein kinase kinase 1 |
| CDH1 | [P12830](http://www.uniprot.org/entry/P12830) | Cadherin-1 |
| MAPK8 | [P45983](http://www.uniprot.org/entry/P45983) | Mitogen-activated protein kinase 8 |
| PDK1 | [Q15118](http://www.uniprot.org/entry/Q15118) | Pyruvate dehydrogenase kinase 1 |
| EIF4G1 | [Q04637](http://www.uniprot.org/entry/Q04637) | Eukaryotic translation initiation factor 4 gamma 1 |
| PIK3C2A | [O00443](http://www.uniprot.org/entry/O00443) | Phosphatidylinositol-4-phosphate 3-kinase catalytic subunit type 2 alpha |
| LCK | [P06239](http://www.uniprot.org/entry/P06239) | LCK proto-oncogene, Src family tyrosine kinase |
| RICTOR | [Q6R327](http://www.uniprot.org/entry/Q6R327) | RPTOR independent companion of MTOR complex 2 |
| PTK2 | [Q05397](http://www.uniprot.org/entry/Q05397) | Protein tyrosine kinase 2 |
| YBX1 | [P67809](http://www.uniprot.org/entry/P67809) | Y-box binding protein 1 |
| GYS1 | [P13807](http://www.uniprot.org/entry/P13807) | Glycogen synthase 1 |
| ARAF | [P10398](http://www.uniprot.org/entry/P10398) | A-Raf proto-oncogene, serine/threonine kinase |
| ELK1 | [P19419](http://www.uniprot.org/entry/P19419) | ETS transcription factor |
| HSBP1 | [O75506](http://www.uniprot.org/entry/O75506) | Heat shock factor binding protein 1 |
| AKT1S1 | [Q96B36](http://www.uniprot.org/entry/Q96B36) | AKT1 substrate 1 |
| NOTCH1 | [P46531](http://www.uniprot.org/entry/P46531) | Notch 1 |
| LDHA | [P00338](http://www.uniprot.org/entry/P00338) | Lactate dehydrogenase A |
| RPS6KA1 | [Q15418](http://www.uniprot.org/entry/Q15418) | Ribosomal protein S6 kinase A1 |
| CREB1 | [P16220](http://www.uniprot.org/entry/P16220) | CAMP responsive element binding protein 1 |
| MS4A1 | [P11836](http://www.uniprot.org/entry/P11836) | Membrane spanning 4-domains A1 |
| PLK1 | [P53350](http://www.uniprot.org/entry/P53350) | Polo like kinase 1 |
| RPS6KA | [Q15418](http://www.uniprot.org/entry/Q15418) | Ribosomal protein S6 kinase A1 |
| MYO2A | P19524 | Myosin type-2 heavy chain 1 |
| TRIM25 | [Q14258](http://www.uniprot.org/entry/Q14258) | Tripartite motif containing 25 |
| PTGS3 | [O09114](http://www.uniprot.org/uniprot/O09114) | Prostaglandin D2 synthase |
